# Mice with AS160/TBC1D4-Thr649Ala Knockin Mutation Are Glucose Intolerant with Reduced Insulin Sensitivity and Altered GLUT4 Trafficking

**DOI:** 10.1016/j.cmet.2010.12.005

**Published:** 2011-01-05

**Authors:** Shuai Chen, David H. Wasserman, Carol MacKintosh, Kei Sakamoto

**Affiliations:** 1 MRC Protein Phosphorylation Unit, College of Life Sciences, University of Dundee, Dow Street, Dundee DD1 5EH, Scotland, UK; 2 Department of Molecular Physiology and Biophysics, Vanderbilt University, School of Medicine, 2200 Pierce Avenue, Nashville, TN 37232, USA

## Abstract

AS160 has emerged as a key player in insulin-mediated glucose transport through controlling GLUT4 trafficking, which is thought to be regulated by insulin-stimulated phosphorylation of sites including the 14-3-3 binding phospho-Thr649 (equivalent to Thr642 in human AS160). To define physiological roles of AS160-Thr649 phosphorylation and 14-3-3 binding in glucose homeostasis, we substituted this residue by a nonphosphorylatable alanine by knockin mutation in mice. The mutant protein was expressed at normal levels, while insulin-stimulated AS160 binding to 14-3-3s was abolished in homozygous knockin mice. These animals displayed impaired glucose disposal and insulin sensitivity, which were associated with decreased glucose uptake in vivo. Insulin-stimulated glucose transport and cell surface GLUT4 content were reduced in isolated muscles, but not in adipocytes. These results provide genetic evidence that insulin-induced AS160-Thr649 phosphorylation and/or its binding to 14-3-3 play an important role in regulating whole-body glucose homeostasis, at least in part through regulating GLUT4 trafficking in muscle.

## Graphical abstract

**Graphical Abstract:**

**Figure:**
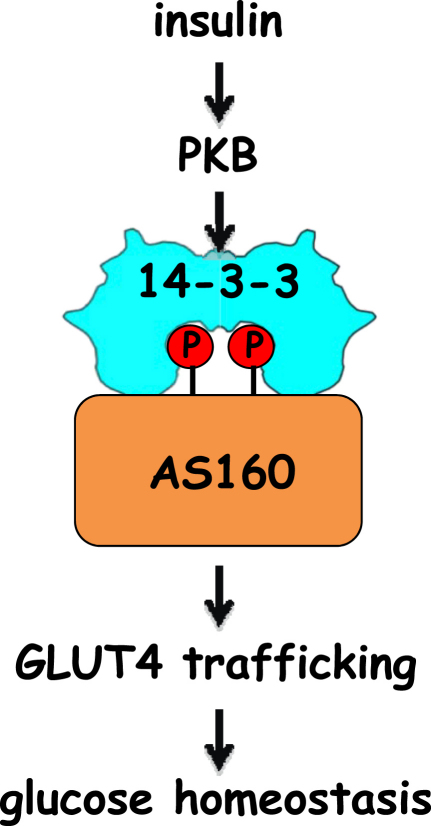

**Highlights:**

► AS160 Thr649→Ala knockin mice are made to study roles of AS160/14-3-3 interaction ► The knockin mice display impaired glucose tolerance and reduced insulin sensitivity ► Deregulated GLUT4 trafficking and glucose uptake in muscles underlie this phenotype ► These findings shed new light on understanding of the pathology of type II diabetes

## Introduction

Insulin promotes uptake of blood glucose mainly into skeletal muscle and fat tissues by mobilizing the glucose transporter GLUT4 from intracellular storage vesicles onto the cell surface where GLUT4 can facilitate the entry of glucose into cells (James et al., 1988). This process involves the engagement of multiple signaling pathways with the exocytosis (or vesicular trafficking) machinery. Numerous studies suggest that the phosphoinositide 3-kinase (PI 3-kinase)/protein kinase B (PKB, also known as Akt) signaling cascade downstream of the insulin receptor plays a central role in controlling insulin-induced GLUT4 translocation (Larance et al., 2008). Though there are three PKB isoforms, only PKBβ (also known as Akt2) is indispensable in controlling GLUT4 trafficking (Cho et al., 2001; Ng et al., 2008).

Recently, the Rab GTPase-activating protein (GAP) termed AS160 (Akt substrate of 160 kDa, also known as TBC1D4) has emerged as a candidate PKB effector for controlling GLUT4 trafficking in fat and muscle cells (Bruss et al., 2005; Eguez et al., 2005; Larance et al., 2005; Sakamoto and Holman, 2008; Sano et al., 2003; Thong et al., 2007; Zeigerer et al., 2004). AS160 contains two N-terminal phosphotyrosine binding domains (PTB), with a RabGAP domain toward the C terminus (Chen et al., 2008; Sano et al., 2003). In unstimulated cells, AS160 is thought to actively maintain its substrate Rab GTPase(s) in a guanosine-5′-diphosphate (GDP)-loaded and inactive form, thereby retaining the GLUT4 storage vesicles within cells (Larance et al., 2005). Upon insulin or insulin-like growth factor (IGF)-1 stimulation of cultured cells, AS160 was found to be phosphorylated at multiple sites including Ser318, Ser341, Ser570, Ser588, Thr642, Ser666, and Ser751, which fall into two clusters flanking the second PTB domain (Geraghty et al., 2007; Sano et al., 2003). In cell-free assays, we showed that PKB directly phosphorylated Ser318, Ser588, and Thr642 (Geraghty et al., 2007). To investigate the role that insulin-stimulated phosphorylation of AS160 has in GLUT4 trafficking, several groups overexpressed an AS160-4P mutant (in which alanine replaces Ser318, Ser588, Thr642, and Ser751) in cultured fat and muscle cells, and found that introduction of 4P mutant efficiently inhibited the insulin-induced increase in the cell surface GLUT4 content (Sano et al., 2003; Thong et al., 2007; Zeigerer et al., 2004). This dominant-negative effect of the 4P mutant has suggested that the insulin-stimulated phosphorylation prevents the GAP activity of AS160 from acting on the downstream Rab(s), which become loaded with guanosine 5′-triphosphate (GTP) and actively promote GLUT4 translocation to cell surface (Randhawa et al., 2008; Sano et al., 2007). Recent genetic analysis identified patients with severe insulin resistance during puberty that carry a premature stop mutation in one allele of AS160 (R363X), resulting in lower levels of AS160 protein together with a dominant-negative truncated variant (Dash et al., 2009). This association of AS160 with human disease heightens the need to define precisely how AS160 regulates glucose homeostasis.

Initially identified as a candidate gene linked to familial female obesity (Stone et al., 2006), TBC1D1 is related to AS160 and has a similar overall molecular architecture (Chen et al., 2008). TBC1D1 is also phosphorylated in two clusters on either side of the second PTB domain (Chavez et al., 2008; Chen et al., 2008; Peck et al., 2009; Taylor et al., 2008). PKB is one of the upstream kinases phosphorylating TBC1D1 at Thr596, which is a paralogue of Thr642 on AS160 (Chen et al., 2008; Roach et al., 2007). Ectopic expression of TBC1D1-3P mutant (in which alanine replaces Ser489, Thr499/Ser501, and Thr590 on mouse TBC1D1 protein) exerted robust inhibition on insulin-induced GLUT4 translocation, indicating that phosphorylation of TBC1D1 also regulates GLUT4 trafficking (Peck et al., 2009).

Phosphorylation of AS160 and TBC1D1 triggers the binding of these proteins to 14-3-3 proteins (Chen et al., 2008; Geraghty et al., 2007; Pozuelo Rubio et al., 2004; Ramm et al., 2006). Insulin and IGF-1 induce 14-3-3 binding to AS160 mainly via phospho-Thr642 with phospho-Ser341 also contributing (Geraghty et al., 2007), whereas activators of AMP-activated protein kinase (AMPK) promote interaction of 14-3-3s with TBC1D1 via its phospho-Ser237 site with phospho-Thr596 playing a secondary role (Chen et al., 2008). The 14-3-3 binding phospho-Thr642 on AS160 is among the four sites mutated in the 4P mutant that can inhibit insulin-induced GLUT4 translocation when overexpressed in muscle and fat cells (Sano et al., 2003; Thong et al., 2007; Zeigerer et al., 2004). Moreover, there is evidence suggesting that 14-3-3 binding to AS160 is required for insulin-stimulated GLUT4 translocation in 3T3-L1 adipocytes (Ramm et al., 2006). We hypothesize therefore that 14-3-3 binding to AS160 regulates whole-body glucose homeostasis in response to insulin, while 14-3-3 binding to TBC1D1 regulates this process in response to insulin and/or AMPK-activating stimuli, such as exercise. Toward addressing this hypothesis, we generated a knockin mouse model in which Thr649 on mouse AS160 (equivalent to Thr642 on human AS160) is mutated to a nonphosphorylatable alanine to prevent 14-3-3 binding in vivo. Here we provide genetic evidence that insulin-mediated phosphorylation (Thr649) and 14-3-3 binding to AS160 plays an important role in regulating whole-body glucose homeostasis, at least in part through regulating GLUT4 trafficking and glucose transport in muscles.

## Results

### Generation and Basic Characterization of AS160 Thr649Ala Knockin Mice

**Figure 1:**
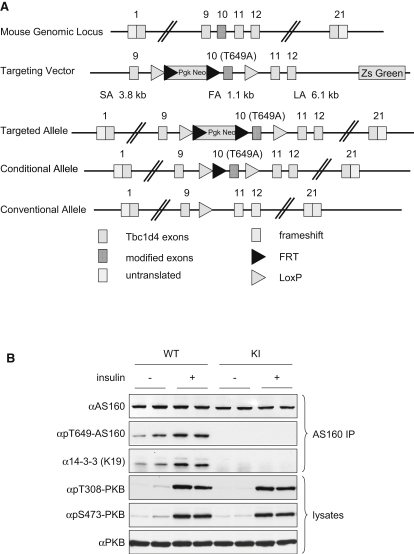
Generation and Basic Characterization of Mice with AS160 Thr649Ala Knockin (A) Strategy for generating mice with AS160 Thr649Ala knockin. The diagram illustrates the targeting knockin construct, the AS160 gene, and the allele modification generated. The Thr649Ala mutation was introduced into exon 10, which is flanked by loxP sites, and the selection marker (Pgk Neo) was flanked by FRT sites. The Thr649Ala knockin was generated after in vivo Flp-mediated removal of selection marker. (B) Thr649 phosphorylation and 14-3-3 binding of AS160. Eight-week-old male mice were anaesthetized and intraperitoneally injected with either saline buffer or insulin (150 mU/g) for 20 min before the respective tissues were removed. In the upper three panels, AS160 proteins were immunoprecipitated from 0.5 mg of heart lysates and Thr649 phosphorylation of AS160 and coprecipitation of 14-3-3s were determined using the Thr649 phosphospecific antibody and K19 14-3-3 antibody, respectively. In the lower three panels, PKB phosphorylation was determined in 40 μg of total heart lysates by using the phosphospecific antibodies recognizing phosphorylated Thr308 and Ser473 on PKB, respectively. KI, knockin; IP, immunoprecipitation. See also Figure S1.

AS160 Thr649Ala knockin mice were generated by using the gene-targeting strategy depicted in Figure 1A. Mating of AS160 Thr649Ala heterozygous (AS160^T649A/+^) animals generated homozygous (AS160^T649A/T649A^), heterozygous, and wild-type (AS160^+/+^) littermate control mice. AS160 protein levels were the same in AS160 knockin and wild-type mice in all the tissues examined, except in the testes where it was modestly reduced in heterozygous and nearly abolished in homozygous knockin mice (Figure S1B, available online). Despite this diminution in AS160 mutant protein level in testes, the homozygous male knockin mice were fertile and the genotypes of progeny were in a Mendelian ratio (data not shown). Insulin had no effect on the amount of total AS160 protein detected in various tissues except in adipose tissue where its levels were increased after insulin administration, regardless of the Thr649Ala mutation (Figure S1B).

**Figure 2:**
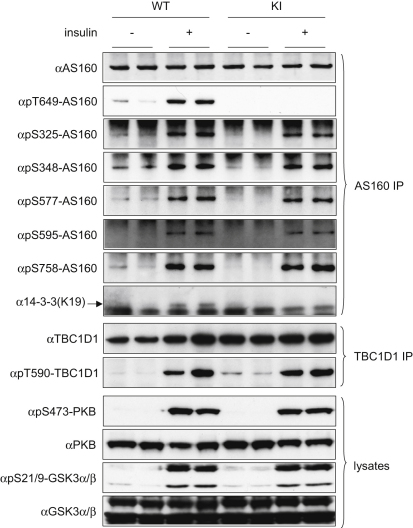
Insulin-Stimulated Phosphorylation of AS160, PKB, GSK3, and TBC1D1 Eight-week-old male homozygous AS160 knockin (KI) mice or their littermate wild-type (WT) animals were anaesthetized and injected with either saline buffer or insulin (150 mU/g) for 20 min before the respective tissues were removed. In the upper eight panels, AS160 proteins were immunoprecipitated from 0.5 mg of gastrocnemius muscle lysates and phosphorylation of AS160 on Ser325, Ser348, Ser577, Ser595, Thr649, and Ser758 and coprecipitation of 14-3-3s were determined by using the phosphospecific antibodies and K19 14-3-3 antibody, respectively. In the middle two panels, TBC1D1 protein was immunoprecipitated from 2 mg of skeletal muscle lysates and its phosphorylation was determined by using the phosphospecific pThr590 antibody. In the lower four panels, the phosphorylation of PKB and GSK3 and total PKB and GSK3 were determined in 40 μg of muscle lysates by using the respective phosphospecific antibodies or total antibodies indicated in the Experimental Procedures. See also Figure S2.

As predicted, because of substitution of threonine 649 to a nonphosphorylatable alanine, neither basal nor insulin-stimulated Thr649 phosphorylation could be detected in tissue extracts from the knockin mice with a phosphospecific pThr649 antibody, in contrast to the insulin-responsiveness of this phosphorylation in extracts from the wild-type animals (Figures 1B and 2 and Figure S2). In parallel with this observation, the insulin-stimulated increase in 14-3-3 binding to AS160 as detected in tissue extracts of wild-type mice was abolished in the knockin animals (Figures 1B and 2 and Figure S2). The insulin-stimulated phosphorylations of the activating sites on PKB (Thr308 and Ser473), a major upstream kinase for AS160, were comparable in both animals (Figures 1B and 2 and Figure S2).

The AS160 homozygous knockin mice were slightly smaller than their wild-type littermates with overall proportional organ sizes (Figures S1C–S1E) though food intake was only marginally affected by the Thr649Ala mutation, consistent with no significant difference in plasma leptin levels (Table 1). Blood glucose, free fatty acid, plasma insulin, and adiponectin levels were not altered in the knockin mice as compared to the wild-type littermates under both fed and fasted states (Table 1).

### AS160 Thr649Ala Knockin Mutation Did Not Cause Detectable Changes in Insulin-Signaling Pathways

The Thr649Ala mutation did not alter the insulin-stimulated phosphorylations of five other sites on AS160, namely Ser325, Ser348, Ser577, Ser595, and Ser758 (residue numbers for the mouse protein; Figure 2 and Figure S2A) in the skeletal and cardiac muscle extracts derived from AS160 knockin animals. The insulin-stimulated phosphorylations of PKB and its substrate GSK3 were also normal in the knockin mice. Moreover, phosphorylation of the activating Thr172 site on AMPK, a master kinase that controls cellular energy metabolism, was not changed in the knockin animals (Figure S2B and data not shown). Similarly, there was no detectable alteration in total amounts and phosphorylation of the key insulin-signaling enzymes in the heart and adipose tissue (Figure S2B and data not shown). Neither the levels of TBC1D1 protein nor the levels of phosphorylation of its insulin-responsive site Thr590 (equivalent to Thr596 on human TBC1D1) were altered in muscles from the knockin mice, indicating that there is no apparent compensatory change in regulation of TBC1D1 protein in AS160 knockin mice (Figure 2). Collectively, these data validate the utility of AS160 knockin mouse for studying the specific function of AS160-Thr649 phosphorylation and 14-3-3/AS160 interaction.

### AS160 Knockin Mice Exhibit Impaired Glucose Tolerance and Reduced Insulin Sensitivity

**Figure 3:**
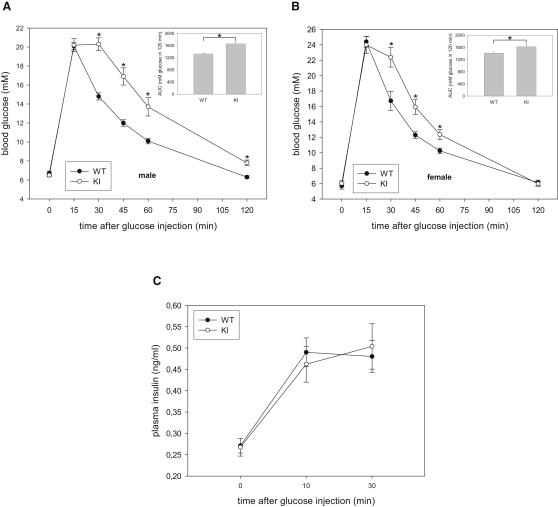
Glucose Clearance and Plasma Insulin Levels in AS160 Knockin Mice after Intraperitoneal Injection with Glucose (A) Glucose tolerance test of 10-week-old male mice. The inset shows the glucose area under the curve (AUC) during glucose tolerance test. The data are given as the mean ± standard error of the mean (SEM) (n = 7–8). Asterisk indicates p < 0.05 (t test). Results shown are representative of four independent experiments with different mouse populations. (B) Glucose tolerance test of 10- to 12-week-old female mice. The inset shows the glucose area under the curve during glucose tolerance test. The data are given as the mean ± SEM (n = 6–7). Asterisk indicates p < 0.05 (t test). (C) Plasma insulin levels during a glucose tolerance test. The values represent the mean (± SEM) from seven to eight male mice (8 weeks old) for each genotype. Results shown are representative of three independent experiments with different mouse populations. See also Figure S3.

**Figure 4:**
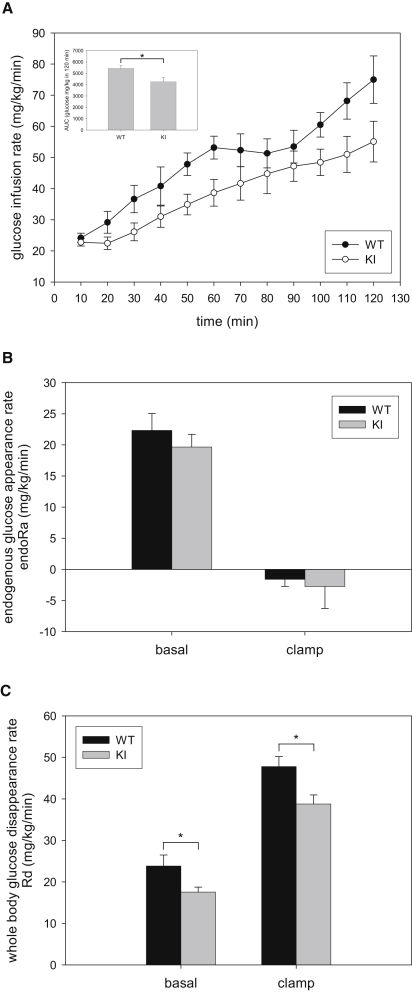
Glucose Infusion Rate, Endogenous Glucose Appearance Rate, and Whole-Body Glucose Disappearance during Hyperinsulinemic-Euglycemic Clamp (A) Glucose infusion rate (GIR) during insulin clamp in AS160 knockin and wild-type male mice (16 weeks old). The inset shows the area under the curve of the GIR during hyperinsulinemic-euglycemic clamp. Data are shown as mean (± SEM) for six to nine mice per genotype. Asterisk indicates p < 0.05 (t test). (B) The endogenous glucose appearance rate (endoR_a_) during basal conditions and during insulin clamps. Basal values are averaged from plasma samples obtained at t = −15 and −5 min prior to onset of insulin clamps. Clamp values are averaged from plasma samples obtained at t = 80–120 min of the insulin clamps. Data are shown as mean (± SEM) for six to nine mice per genotype. (C) Whole-body glucose disappearance (R_d_) during basal conditions and during insulin clamps. Basal values are averaged from plasma samples obtained at t = −15 and −5 min prior to onset of insulin clamps. Clamp values are averaged from plasma samples obtained at t = 80–120 min of the insulin clamps. Data are shown as mean (± SEM) for six to nine mice per genotype. Asterisk indicates p < 0.05 (t test). See also Figure S4.

Because AS160 has been implicated in insulin-stimulated glucose transport in muscle and fat cells (Sano et al., 2003; Thong et al., 2007) and also in glucose-induced insulin secretion from pancreatic beta cells (Bouzakri et al., 2008), we sought to determine if whole-body glucose homeostasis is altered in the knockin animals. We initially observed that levels of blood glucose and plasma insulin in overnight-fasted and randomly-fed states were comparable between knockin and wild-type mice (Table 1). We then performed glucose tolerance test and injected a bolus of glucose (intraperitoneal [i.p.], 2 mg/g body weight), which led to similar increases in blood glucose levels in both knockin and wild-type mice 15 min after the injection. Strikingly, however, there was a marked delay in clearance of blood glucose in the knockin animals (both males and females) compared with wild-type littermates (Figures 3A and 3B). The areas under the blood glucose response curve (AUC) were significantly increased by 25% and 14% in the knockin male and female mice, respectively, as compared with the wild-type littermates (Figures 3A and 3B). These results demonstrate that the AS160 knockin mice are glucose intolerant. To check whether impaired glucose tolerance observed in the knockin animals was due to defects in insulin secretion and/or clearance, we assessed plasma insulin levels during glucose tolerance test. We observed that the glucose-induced increase in plasma insulin levels was similar between knockin and wild-type mice within the first hour (10, 30, 45, and 60 min) after glucose injection (Figure 3C and data not shown), indicating that altered insulin levels in the blood were unlikely to be responsible for the glucose intolerance phenotype of the knockin mice. We next injected a bolus of intermediate insulin dose (0.75 mU/g body weight) to assess whether or not AS160 knockin mice are insulin resistant. Although there was an apparent trend toward moderate blunting of insulin-induced hypoglycemia in the knockin animals, the difference was not statistically significant compared to the wild-type mice (Figure S3). To investigate whole-body insulin sensitivity in a more physiological context (avoiding counterregulatory responses induced by hypoglycemia in insulin tolerance tests), we carried out a hyperinsulinemic-euglycemic clamp study. Blood glucose levels were tightly maintained through a variable glucose infusion rate (GIR) in response to a constant insulin infusion in both genotypes (Figures S4A and S4B). The GIR required to maintain euglycemia (∼5.5 mM) was significantly lower in the knockin mice than the wild-type littermates (Figure 4A), indicating that the knockin mice were insulin resistant (Figures 3A and 3B). Endogenous glucose production through hepatic gluconeogenesis and renal glucose reabsorption are unlikely to account for the reduced insulin sensitivity during the clamp, as the rates of endogenous glucose appearance (endoR_a_) in the basal state were comparable and insulin infusion fully suppressed endoR_a_ in both genotypes (Figure 4B). We also observed that in response to glucose injection (i.p., 2 mg/g body weight), the livers of the knockin mice and their wild-type littermates displayed similar phosphorylation of PKB, dephosphorylation of liver glycogen phosphorylase, which mediates inhibition of glycogenolysis, and dephosphorylation of glycogen synthase, which activates glycogen synthesis (Figure S4E). Furthermore, the knockin and wild-type mice also exhibited identical blood glucose profiles in response to pyruvate challenge (Figure S4F), indicating that hepatic gluconeogenesis from pyruvate was unaffected. Notably, the rate of whole-body glucose disappearance (R_d_) was significantly lower in the knockin mice than their wild-type littermates during the clamp (Figure 4C). Taken together, it would appear that decreased glucose disposal was the likely underlying cause for the insulin resistance and glucose intolerance in the AS160 knockin mice.

However, several issues must be considered in the interpretation of the hyperinsulemic-euglycemic clamp experiments. First, arterial plasma insulin levels at the end of clamp tended to be reduced in AS160 knockin mice (Figure S4B). While not approaching significance (p = 0.128), this reduction could conceivably contribute to the reduction in glucose utilization. However, we found no correlation between endpoint insulin levels and R_d_ (R^2^ = 0.0969, Figure S4D). Furthermore, we confirmed that lower endpoint insulin levels in the knockin mice were not associated with reduced GIR (data not shown). Thus, the tendency for reduced arterial insulin levels at the end of clamp has no apparent statistical or physiological significance. In addition, in our experience the biological variation of arterial insulin in the short-term fasted conscious mouse is greater than in other species and insulin concentrations during a clamp in the mouse are variable (Ayala et al., 2006; Berglund et al., 2008).

The GIR during the hyperinsulinemic-euglycemic clamp did not achieve a steady state but continued to rise (Figure 4A), suggesting that insulin action did not obtain a steady state. This is in contrast with other studies from our laboratory that show a steady state is achieved in this time frame (Ayala et al., 2006). Changes in the GIR will have no impact on the total rate of glucose appearance and disappearance in the nonsteady state, provided that the glucose-specific activity is stable. Figure S4C shows that glucose-specific activity was indeed stable during the tracer-sampling period (t = 80 to 120 min). The calculation of endogenous glucose production is the difference between the total glucose appearance rate and the GIR. The instability of the GIR was paralleled by a decline in the endoR_a_ and resulted in negative rates of endoR_a_ (Figure 4B). Again, it is important to emphasize that the change in the GIR per se has no impact on the nonsteady-state calculation of the rate of glucose disappearance and does not impact conclusions regarding muscle glucose utilization, which is the focus of these experiments.

### Altered Glucose Transport in Isolated Skeletal Muscle and Primary Adipocytes from AS160 Knockin Mice

**Figure 5:**
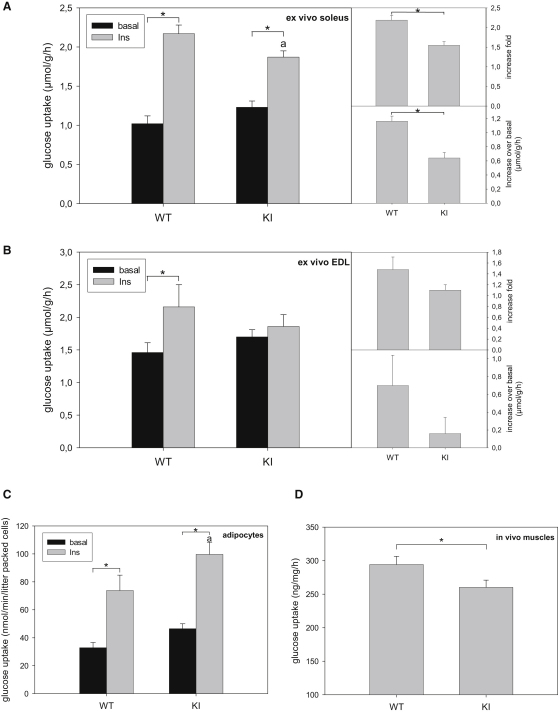
Insulin-Stimulated Glucose Transport in Ex Vivo Muscles and Primary Adipocytes (A) Insulin-stimulated glucose transport in ex vivo soleus muscles. The soleus muscles were isolated from male mice (10–12 weeks old) stimulated with or without 0.1 mU/ml of insulin and used for glucose transport assay. A fold increase of the rates of glucose transport and an increase of the rates of glucose transport upon insulin stimulation over the basal state were deduced from the glucose transport assay. The values are given as the mean ± SEM (n = 7). Asterisk indicates p < 0.05. a indicates p < 0.05 versus WT (insulin). (B) Insulin-stimulated glucose transport in the ex vivo EDL muscles. The EDL muscles were isolated from both wild-type and AS160 knockin mice (8–9 weeks old) stimulated with or without 0.1 mU/ml of insulin and used for glucose transport assay. A fold increase of the rates of glucose transport and an increase of the rates of glucose transport upon insulin stimulation over the basal state were deduced from the glucose transport assay. Asterisk indicates p < 0.05. (C) Insulin-stimulated glucose transport in primary adipocytes. The primary adipocytes were isolated from the epididymal fat pads of male mice (10 weeks old) stimulated with or without 16.7 mU/ml insulin for 30 min and used for glucose transport assay. The values are given as the mean ± SEM (n = 8). Asterisk indicates indicates p < 0.05. a indicates p < 0.05 versus WT (insulin). (D) In vivo muscle glucose uptake. Male mice at 12 to 14 weeks of age were subject to intraperitoneal injection of glucose (2 mg/g body weight) containing 2-deoxy-[^3^H]-glucose. At 60 min after injection, both tibialis anterior and quadriceps muscles were removed and used to determine muscle glucose uptake. The values are given as the mean ± SEM (n = 5). ^∗^ indicates p < 0.05 (t test).

To deduce the mechanisms underlying the decreased glucose disposal and insulin sensitivity in AS160 knockin mice, we measured glucose transport in isolated skeletal muscles and primary adipocytes ex vivo. For soleus, glucose uptake into the knockin muscles was slightly higher than wild-type muscles under unstimulated conditions, but in contrast was significantly lower (∼15%) for knockin muscles compared with wild-type muscles upon insulin stimulation (Figure 5A). Overall, both the fold increase in glucose transport upon insulin and the ΔRate_Insulin-Basal_ were significantly lower in soleus from the knockin mice (Figure 5A). Similar results were also obtained in the isolated extensor digitorum longus (EDL) muscles (Figure 5B), although the ability of insulin to stimulate glucose uptake in EDL was much more modest under our experimental conditions than in soleus, as previously reported (Sakamoto et al., 2005). We confirmed that insulin robustly stimulated PKB to the same extent in ex vivo muscles from both genotypes, whereas AS160-Thr649 phosphorylation was only potently stimulated by insulin in muscles from the wild-type mice, but not from knockin mice (data not shown).

Under basal conditions, glucose uptake into primary adipocytes from AS160 knockin mice was slightly higher than into wild-type cells (Figure 5C). However, unlike the isolated muscles, insulin stimulated a significantly higher glucose uptake into primary adipocytes from the knockin mice compared with cells from the wild-type littermates (Figure 5C), resulting in a comparable fold increase in glucose uptake upon insulin treatment between the two genotypes.

### AS160 Knockin Mice Have a Lower Rate of Glucose Uptake into Skeletal Muscles In Vivo

To address the physiological relevance of the reduced insulin-stimulated glucose uptake observed in isolated muscles, we measured muscle glucose transport in vivo by injection of glucose (i.p., 2 mg/g body weight) containing 2-deoxy-[^3^H]-glucose into mice. We observed that glucose uptake into hindlimb skeletal muscles from the knockin mice was modestly, but significantly (∼12%) lower than from the wild-type littermates (Figure 5D). This observation together with ex vivo muscle glucose uptake data strongly suggest that the reduced rate of insulin-mediated uptake of glucose into muscles is the major cause underlying the glucose intolerance phenotype in the knockin mice.

### Impaired GLUT4 Translocation from Intracellular Compartments onto Plasma Membrane in AS160 Knockin Mice

**Figure 6:**
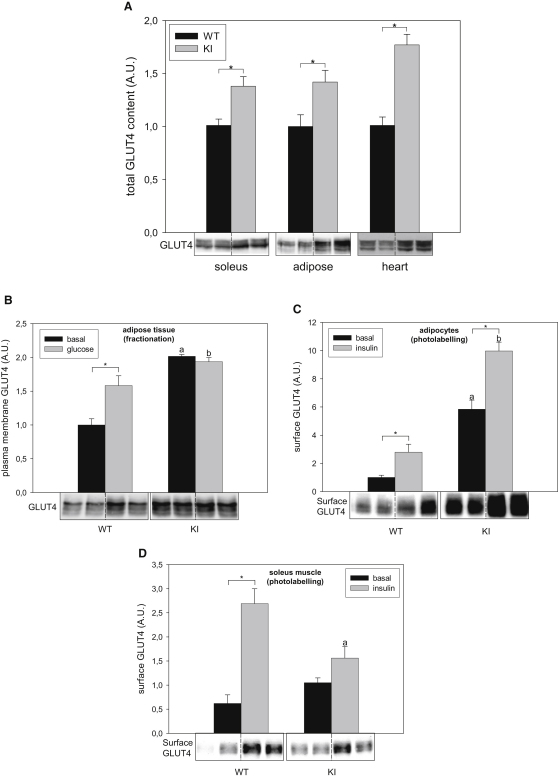
The Total GLUT4 Protein Levels and GLUT4 Translocation in the Wild-Type and AS160 Knockin Mice (A) The total GLUT4 protein levels in AS160 knockin and wild-type mice (8 weeks old). GLUT4 proteins were detected in 40 μg of total lysates of soleus muscles, adipose tissue, and hearts by using anti-GLUT4 antibody and the signals were quantified. The data are given as the mean ± SEM (n = 8–9). Asterisk indicates p < 0.05 (t test). Representative blots are shown. (B) Plasma membrane-bound GLUT4 in adipose tissue from male mice (10–12 weeks old) after intraperitoneal injection of glucose or saline. The mice that were fasted for 16 hr were subjected to intraperitoneal injection of glucose (2 mg/g body weight) or saline and tissues were taken at 20 min after injection. The plasma membrane fractions were separated from the intracellular fractions containing GLUT4 vesicles. GLUT4 levels were determined via western blot and the signals were quantified (n = 3 per genotype per treatment). The data were given as the mean ± SEM. Asterisk indicates p < 0.05; a indicates p < 0.05 versus WT (basal); b indicates p < 0.05 versus WT (glucose). A representative blot is shown. (C) Cell surface GLUT4 levels in primary adipocytes. Primary adipocytes were isolated from male mice (15–16 weeks old). The primary adipocytes were isolated from the epididymal fat pads and stimulated with or without 0.17 mU/ml of insulin for 30 min. Surface GLUT4 was then chemically tagged with the Bio-LC-ATB-BGPA, pulled down by using Streptavidin beads from equal amounts of lysates, and quantified via western blot. The data were given as the mean ± SEM (n = 4). ^∗^ indicates p < 0.05. a, p < 0.05 versus WT (basal); b, p < 0.05 versus WT (insulin). A representative blot is shown. (D) Cell surface GLUT4 levels in the ex vivo soleus muscles. The soleus muscles were isolated from wild-type and AS160 knockin mice (8–12 weeks old), stimulated or not with 0.1 mU/ml of insulin and used for photolabelling assay. Surface GLUT4 was then chemically tagged with the Bio-LC-ATB-BGPA, pulled down from equal amounts of lysates by using Streptavidin beads, and quantified via western blot. The data were given as the mean ± SEM (n = 4–5). ^∗^ indicates p < 0.05; a indicates p < 0.05 versus WT (insulin). A representative blot is shown. See also Figure S5.

To define the mechanism underlying the altered glucose uptake of muscles and primary adipocytes we examined expression of GLUT4, which is the major insulin-regulated glucose transporter mediating insulin-induced uptake of glucose into heart, skeletal muscles, and adipose tissue. Interestingly, GLUT4 protein levels in extracts of these tissues were higher for the knockin mice than the wild-type mice (Figure 6A; data not shown). However, *glut*4 transcript levels were not significantly altered (Figure S5A), indicating that the mechanism(s) underlying the increase of GLUT4 protein levels are posttranscriptional.

GLUT4 translocation from intracellular storage compartments onto plasma membrane was determined via membrane fractionation in adipose tissue and skeletal muscles from the mice that had been injected with glucose (i.p., 2 mg/g body weight) to increase blood insulin levels within the physiological range. The presence of the plasma membrane in the pelleted fraction was established by measuring the distribution of the Na^+^/K^+^-ATPase α-1 subunit and insulin receptor β subunit between the pelleted fraction and the cytosol fraction (Figure S5B). The cytosol fraction should contain the intracellular GLUT4 vesicles, because previous studies have shown that the vesicles are not pelleted under the conditions used here (Larance et al., 2005). Consistent with the elevated GLUT4 levels, both plasma membrane-associated and intracellular GLUT4 levels were higher in the knockin mice (Figure 6B and data not shown). Intriguingly, glucose injection increased plasma membrane-associated GLUT4 only in the adipose tissue (1.6-fold) from the wild-type mice, but not from AS160 knockin animals (Figure 6B). We confirmed that the inability of glucose administration to promote GLUT4 translocation in the knockin mice was not due to reduced PKB activation (data not shown). Although we observed that glucose injection slightly increased the amounts of plasma membrane-bound GLUT4 in the muscles from the wild-type mice but not the knockin animals (data not shown), we were not able to draw a decisive conclusion because of technical difficulties in preparing pure plasma membrane with high yield from this tissue.

To further establish the impaired recruitment of GLUT4 to the plasma membrane, we quantitatively measured cell-surface-exposed GLUT4 protein by a photolabelling method (Holman et al., 1990) in primary adipocytes and isolated soleus muscles. In the primary adipocytes, surface GLUT4 levels at basal states were elevated by nearly 6-fold in the knockin cells (Figure 6C) in contrast to only a 50% increase in the basal glucose transport rates (Figure 5C), as compared with wild-type cells. Stimulation with insulin led to a nearly 3-fold increase in surface GLUT4 levels in wild-type cells (Figure 6C). Interestingly, while insulin had not changed the cytoplasmic-to-plasma-membrane-associated partitioning of GLUT4 in knockin adipocytes (Figure 6B and data not shown), insulin did increase the amount of surface-exposed GLUT4 by nearly 2-fold (Figure 6C). In resting soleus muscles, surface GLUT4 levels were slightly higher, though not statistically significant, in the knockins than in the wild-types (Figure 6D). In contrast, surface GLUT4 levels were significantly lower in the knockin soleus than the wild-type muscles upon insulin stimulation (Figure 6D), which is consistent with the glucose transport rates (Figure 5A), suggesting that impaired GLUT4 surface gain might account for the lower insulin-stimulated glucose transport rates in the muscles from the knockin mice.

## Discussion

Here, we generated a genetic knockin mouse model to study the specific roles that phospho-Thr649 and its resulting binding of 14-3-3 to AS160 play in insulin-mediated GLUT4 trafficking and glucose homeostasis. The most striking metabolic phenotype in AS160 knock-in mice (8 to 16 weeks old) is that they display reduced glucose disposal and whole-body insulin sensitivity. These mice are unable to absorb glucose from the blood as efficiently as the wild-type animals in response to insulin and glucose challenge, even though they had elevated levels of GLUT4 relative to wild-type animals in muscle and adipose tissues.

Overall, our data indicate that loss of Thr649 phosphorylation and/or 14-3-3 binding of AS160 impacts on glucose homeostasis by: (1) increasing the expression of GLUT4 proteins through an unknown mechanism that does not involve a change in steady-state *glut4* transcript levels, (2) decreasing the insulin-induced surface GLUT4 expression and glucose transport rates in isolated muscles ex vivo and in intact muscles in vivo, (3) causing a lag in clearance of blood glucose in response to glucose load, and (4) impairing insulin sensitivity in hyperinsulinemic-euglycemic clamp with lower glucose disposal rates.

The glucose intolerance in AS160 knockin mice was not associated with changes in the levels of plasma insulin or adiponectin, but with a lower rate of insulin-stimulated glucose uptake into muscles. Elevating insulin levels in blood after glucose challenge not only stimulates glucose transport into muscle and fat tissues but also suppresses hepatic glucose production and deregulation of either process can lead to glucose intolerance. Hyperinsulinemia during the clamp study fully suppressed hepatic glucose production in AS160 knockin mice as indicated by the ablation of endoR_a_ (Figure 4B). These data suggest that enhanced hepatic gluconeogenesis is unlikely to be the underlying cause for the glucose intolerance and insulin resistance phenotypes observed in the knockin mice. The R_d_ that is mainly determined by muscles in the hyperinsulinemic-euglycemic clamp was significantly lower in the knockin mice than their wild-type littermates (Figure 4C), suggesting that the glucose intolerance in the knockin mice was mainly caused by reduced insulin-mediated glucose transport in muscle. This was supported by our results that AS160 knockin mice exhibit a reduced rate of glucose uptake in muscle in vivo after glucose injection (Figure 5D) and a lesser response to insulin of glucose uptake into isolated soleus and EDL muscles from the knockin animals ex vivo (Figures 5A and 5B). At the molecular level, the knockin mutation decreased the insulin-induced cell surface GLUT4 expression in the muscles from the knockin mice (Figure 6D), which would be expected to consequently limit the insulin-stimulated glucose transport.

While our data are internally consistent in general and supportive of AS160 phosphorylation and/or 14-3-3 binding mediating insulin-stimulated glucose uptake into target tissues, there are several mismatches that we cannot explain, which point to further unknown regulatory steps in glucose transport. First, there is a clear discrepancy in finding that the R_d_ in the insulin clamp study was significantly lower in knockins than wild-types under basal conditions (Figure 4C), and yet the glucose transport rates were moderately higher in unstimulated isolated muscles and primary adipocytes from the knockin mice ex vivo (Figure 5). We cannot rule out the possibility that ex vivo systems using isolated muscle or adipocytes do not always mimic the physiological in vivo regulation of glucose transport in mice mainly because of the absence of various contributing factors (e.g., systemic and neural factors). It is also possible that AS160 plays some additional roles in other tissues that affect glucose metabolism in vivo. Another discrepancy is between surface GLUT4 levels and glucose transport rates in primary adipocytes. The surface GLUT4 level was elevated by nearly 6-fold in the knockin adipocytes relative to wild-type cells (Figure 6C) in contrast to only a 50% increase in basal glucose transport rate (Figure 5C), suggesting that the surface GLUT4 in knockin cells was not as active as the GLUT4 in wild-type cells. Consistent with this suggestion, the magnitude of insulin-stimulated surface GLUT4 expression approximated the fold increase in glucose transport rates in both knockin and wild-type primary adipocytes even though the knockin cells already had much more GLUT4 at the plasma membrane. There is a precedent for surface GLUT4 expression being dissociated from glucose transport, in which a cell-permeable phosphoinositide-binding peptide increased surface GLUT4 levels without stimulating glucose uptake in 3T3-L1 adipocytes (Funaki et al., 2006). It has been proposed therefore that surface GLUT4 needs an activation step, mediated by phosphatidylinositol-4,5-bisphosphate, to be fully functional (Funaki et al., 2006). Moreover, optimal activation of glucose transport in cardiomyocytes upon insulin stimulation requires supportive events such as insulin-induced cytosol alkalinization through the Na^+^/H^+^ exchanger and H^+^-ATPase (Yang et al., 2002). Thus, we speculate that the activation of GLUT4 and/or the supportive events for optimal activation of glucose transport might be impaired or “oversaturated” by elevated GLUT4 levels in AS160 knockin mice, contributing to the lower R_d_ in the knockin mice.

The third paradox centers on the trafficking of GLUT4 from plasma membrane to cell surface. Insulin-induced GLUT4 translocation from intracellular storage vesicles onto cell surface involves multiple stages including soluble N-ethylmaleimide-sensitive factor attachment protein receptor (SNARE)-mediated docking and fusion steps (Hou and Pessin, 2007). The AS160-4P mutant has been found to inhibit insulin-induced GLUT4 translocation in 3T3-L1 adipocytes as well as L6 muscle cells (Sano et al., 2003; Thong et al., 2007; Zeigerer et al., 2004). Recent studies with overexpression of the AS160-4P mutant in 3T3-L1 adipocytes by using total internal reflection fluorescence microscopy (TIRFM) suggest that AS160 regulates the docking step but not the postdocking steps such as fusion of GLUT4 storage vesicles (Bai et al., 2007; Jiang et al., 2008). Consistent with this notion, our data on membrane fractionation show recruitment of GLUT4 from intracellular compartments onto plasma membrane to be impaired in AS160 knockin mice (Figure 6B and data not shown), indicating that AS160-Thr649 phosphorylation and/or 14-3-3 binding play an important role in regulating GLUT4 trafficking. And yet, cell surface GLUT4 expression (determined via the photolabelling assay) and glucose uptake in knockin primary adipocytes and isolated muscles were still stimulated by insulin (Figures 5 and 6), albeit with an overall lower ± insulin ratio in knockin muscles compared with the wild-type tissues. It is worthwhile to point out that membrane fractionation measures plasma membrane-bound GLUT4 including both docked and surface-exposed GLUT4, whereas the photolabelling assay determines only surface-exposed GLUT4. One possible explanation for the discrepancy between GLUT4 levels determined by membrane fractionation and photolabelling assay is that insulin also stimulates a final plasma membrane-fusion step in GLUT4 trafficking (Jiang et al., 2008). In the knockin mice, both the total and plasma membrane-bound pools of GLUT4 were elevated, presumably providing a larger than normal pool of GLUT4 in vesicles that have docked with plasma membrane and are poised and ready to undergo a final AS160-Thr649 phosphorylation-independent fusion step in response to insulin. Consequently, the pool of plasma membrane-bound GLUT4 could partially compensate for inhibition of the docking step imposed by the AS160 Thr649Ala mutation, resulting in an insulin-induced surface GLUT4 expression and glucose transport in ex vivo muscles and primary adipocytes. However, the glucose intolerance and insulin resistance whole-body phenotypes of AS160 knockin mice indicates that this compensatory response cannot completely overcome the impaired GLUT4 trafficking from intracellular storage sites to plasma membrane caused by the AS160 Thr649Ala mutation. Although our data suggest that AS160 Thr649Ala mutation affects GLUT4 trafficking, the exact site in GLUT4 translocation regulated by AS160-Thr649 phosphorylation and/or 14-3-3 binding is still to be identified. One idea is to cross the AS160 knockin mouse with the transgenic mouse expressing green fluorescent protein (GFP) and hemagglutinin (HA)-epitope-tagged GLUT4 that has recently been generated (Fazakerley et al., 2009). More detailed dissection of the GLUT4 trafficking in the offspring, taking advantage of TIRFM, should give further insights into the mechanisms of how AS160-Thr649 phosphorylation and 14-3-3 binding regulate GLUT4 trafficking.

## Experimental Procedures

### Hyperinsulinemic-Euglycemic Clamp

Male mice at 16 weeks of age were used for a hyperinsulinemic-euglycemic clamp study via tail sampling, performed at the NIH Mouse Metabolic Phenotyping Center and led by David Wasserman (Vanderbilt University), as previously described (Ayala et al., 2006). Whole-body R_a_ and R_d_ were determined by using Steele nonsteady-state equations (Steele et al., 1956). The endoR_a_ was determined by subtracting the GIR from total R_a_.

### Glucose Uptake in Isolated Soleus and EDL Muscles and Primary Adipocytes

For glucose uptake assays in isolated muscles ex vivo, mice were killed by cervical dislocation after overnight fast (16 hr). Soleus and EDL muscles were isolated and 2-deoxy-[^3^H]glucose uptake was measured as described (Sakamoto et al., 2005). Briefly, muscles were incubated in 8 ml Krebs-Ringer bicarbonate (KRB) buffer (117 mM NaCl, 4.7 mM KCl, 2.5 mM CaCl_2_, 1.2 mM KH_2_PO_4_, 1.2 mM MgSO_4_, 24.6 mM NaHCO_3_, pH 7.4) containing 2 mM pyruvate with or without insulin (0.1 mU/ml) for 50 min at 37°C. Muscles were transferred to 2 ml KRB containing 1 mM 2-deoxy-D-[1,2-^3^H(N)]glucose (3 μCi) and 7 mM D-[1-^14^C]mannitol (0.9 μCi) and incubated for an additional 10 min with or without insulin at 30°C. Incubation and transport buffers were continuously gassed with 95% O_2_-5% CO_2_. Transport was terminated by immersion in ice-cold KRB containing 80 μM cytochalasin B. Muscles were frozen in liquid nitrogen and processed as previously described (Sakamoto et al., 2005).

For glucose uptake in primary adipocytes, mice were killed by cervical dislocation after overnight fast (16 hr) and epididymal fat was rapidly removed. Primary adipocytes were isolated and glucose transport assay was carried out in primary adipocytes as described previously (Holman et al., 1990). Briefly, epididymal fat was minced and digested in Krebs-Ringer-4-(2-hydroxyethyl)-1-piperazineethanesulfonic acid (HEPES) buffer (KRH; 140 mM NaCl, 4.7 mM KCl, 2.5 mM CaCl_2_, 1.25 mM MgCl_2_, 2.5 mM NaH_2_PO_4_, 10 mM HEPES, pH 7.4) containing 1 mg/ml collagenase (Worthington, Type I), 3.5% (w/v) bovine serum albumin (BSA), 5 mM glucose, and 200 nM adenosine at 37°C for 40 min. The resulting cell suspension was filtered through a nylon mesh (400 μm mesh size, Lockertex) and washed three times with 2.5% (w/v) BSA/KRH buffer containing 200 nM adenosine. The cell suspension was adjusted to a cytocrit of ∼20% and incubated with or without 16.7 mU/ml of insulin at 37°C for 30 min. The adipocytes were incubated in 2.5% (w/v) BSA/KRH buffer containing 40 μM 2-deoxy-D-[U-^14^C]glucose (0.6 μCi) at 37°C for 30 min in the presence or absence of 16.7 mU/ml of insulin. After stopping glucose transport by adding cytochalasin B (30 μM), cell suspension was centrifuged through dinonylphthalate oil and used for scintillation counting.

### Photolabelling of Cell Surface GLUT4 in Isolated Soleus Muscles and Primary Adipocytes

Photolabelling of cell surface GLUT4 with a biotinylated photolabeling reagent, Bio-LC-ATB-BGPA, in primary adipocytes (Holman et al., 1990) and isolated muscle (Karlsson et al., 2009) was carried out as previously described. Briefly, primary adipocytes were stimulated with insulin (0.17 mU/ml) or not after isolation from epididymal fat pads. The cells were then incubated with 200 μM of Bio-LC-ATB-BGPA at room temperature for 8 min and irradiated under UV light for 1 min with a Rayonet Photochemical Chamber Reactor (Southern New England Ultra Violet Company) to crosslink GLUT4 with the photolabels. After crosslinking, the cells were lysed and subjected to further analyses. Isolated soleus muscles were first incubated in the presence or absence of insulin (0.1 mU/ml) for 50 min at 37°C and then for 8 min at room temperature. After incubation with 200 μM Bio-LC-ATB-BGPA at room temperature for 8 min, the muscles were irradiated twice under UV light for 3 min each time. The muscles were snap-frozen after being rinsed. The GLUT4 proteins chemically tagged with photolabels were isolated by using Streptavidin beads and subsequently detected and quantified via immunoblotting analysis.

### Measurement of Muscle Glucose Uptake In Vivo

Skeletal muscle glucose uptake in vivo was measured as previously described with some modification (Bouskila et al., 2008; Kramer et al., 2006). Briefly, mice were fasted overnight and then anesthetized with sodium pentobarbital diluted in saline buffer (90 mg/kg body weight). After ∼15 min blood was taken from the tail to assess basal glucose and background radioactivity levels. A bolus of 2 mg D-glucose per body weight containing 2-deoxy-[^3^H]glucose (10 μCi per ∼20–25 g mouse) was administered via an i.p. injection and blood samples were taken 15, 30, 45, and 60 min later for the determination of blood glucose and 2-deoxy-[^3^H]glucose-specific activity. After the last blood draw, animals were sacrificed by cervical dislocation, the tibialis anterior and gastrocnemius muscles were removed, and the muscles were immediately frozen in liquid nitrogen. Accumulation of 2-deoxy-[^3^H]glucose-6-phosphate from one muscle was assessed via a precipitation protocol as previously described (Ferré et al., 1985) by using barium hydroxide, zinc sulfate, and perchloric acid.

## Figures and Tables

**Table 1:** Basic Characterization of AS160/TBC1D4-Thr649Ala Knockin Mice

	Fasted		Fed	
	WT	KI	WT	KI
Insulin (ng/ml)	0.271 ± 0.017	0.268 ± 0.021	1.014 ± 0.150	1.056 ± 0.122
Blood glucose (mM)	6.7 ± 0.2	6.5 ± 0.2	10.5 ± 0.4	10.0 ± 0.5
Adiponectin (ng/ml)	42.90 ± 1.14	45.50 ± 1.76	42.96 ± 1.61	47.30 ± 1.39
Leptin (ng/ml)	1.799 ± 0.198	1.152 ± 0.128	n.d.	n.d.
FFA (mM)	0.983 ± 0.117	0.924 ± 0.052	n.d.	n.d.

Plasma insulin, blood glucose, adiponectin, leptin, and FFA concentrations were determined in 10 to 12-week-old male mice after fasting (5 hr in the case of adiponectin, leptin, and FFA; 16 hr for plasma insulin and blood glucose measurements) and randomly fed conditions. Values are given as the mean ± SEM from at least seven animals. FFA, free fatty acids; KI, knockin; n.d., not determined; SEM, standard error of the mean; WT, wild-type.

## Supplemental Information

Document S1. Five Figures and Supplemental Experimental Procedures
