# Supplementary material for: Mice with AS160/TBC1D4-Thr649Ala Knockin Mutation Are Glucose Intolerant with Reduced Insulin Sensitivity and Altered GLUT4 Trafficking
Source: Cell Metab. 2011 Jan 5;13(1):68–79. doi: 10.1016/j.cmet.2010.12.005 (PMC3081066; doi:10.1016/j.cmet.2010.12.005)
Supplement: Document S1. Five Figures and Supplemental Experimental Procedures [file mmc1.pdf]

Cell Metabolism, Volume *13*

Supplemental Information

Mice with AS160/TBC1D4-Thr649Ala Knockin Mutation Are Glucose Intolerant with Reduced Insulin Sensitivity and Altered GLUT4 Trafficking

Shuai Chen, David H. Wasserman, Carol MacKintosh, and Kei Sakamoto

Figure S1A

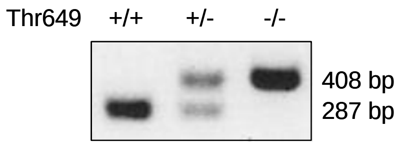

Figure S1B

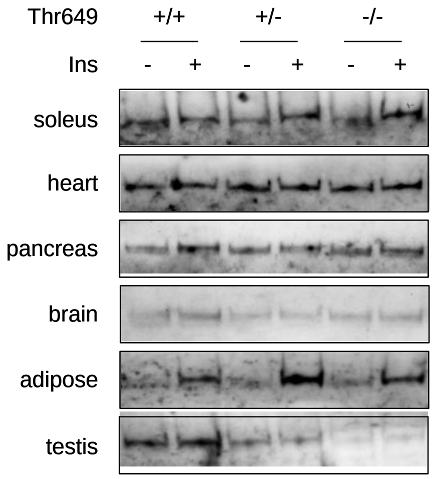

Figure S1C

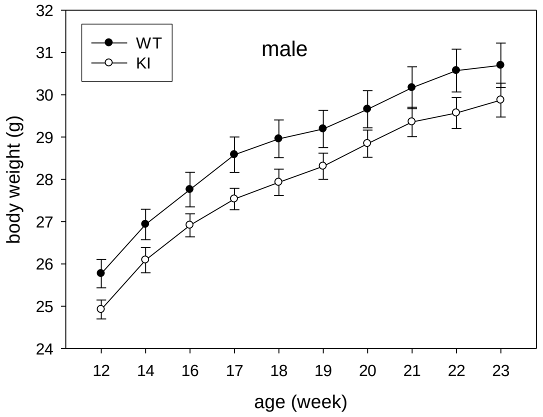

Figure S1D

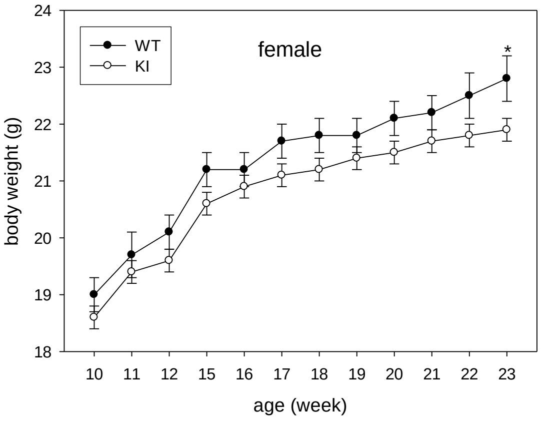

Figure S1E

	Male		Female	
	WT	KI	WT	KI
body weight (g)	31.3 ± 0.8	28.4 ± 0.7*	23.7 ± 0.4	21.7 ± 0.7*
heart/bw (mg/g)	4.40 ± 0.10	4.79 ± 0.15*	4.88 ±0.11	5.16 ± 0.13
Skeletal muscle (tibialis anterior)/bw (mg/g)	1.50 ± 0.04	1.58 ± 0.04	1.63 ± 0.02	1.66 ± 0.04
testis/bw (mg/g)	3.37 ± 0.15	3.80 ± 0.11*	-	-
kidney/bw (mg/g)	5.00 ± 0.13	5.08 ± 0.15	5.15 ± 0.06	5.06 ± 0.12
spleen/bw (mg/g)	2.96 ± 0.41	2.78 ± 0.27	3.98 ± 0.19	3.20 ± 0.31*
liver/bw (mg/g)	39.66 ± 1.16	39.07 ± 0.68	41.60 ± 1.88	43.97 ± 1.30
epididymal fat/bw (mg/g)	18.75 ± 2.24	17.74 ± 1.62	-	-

Figure S2A

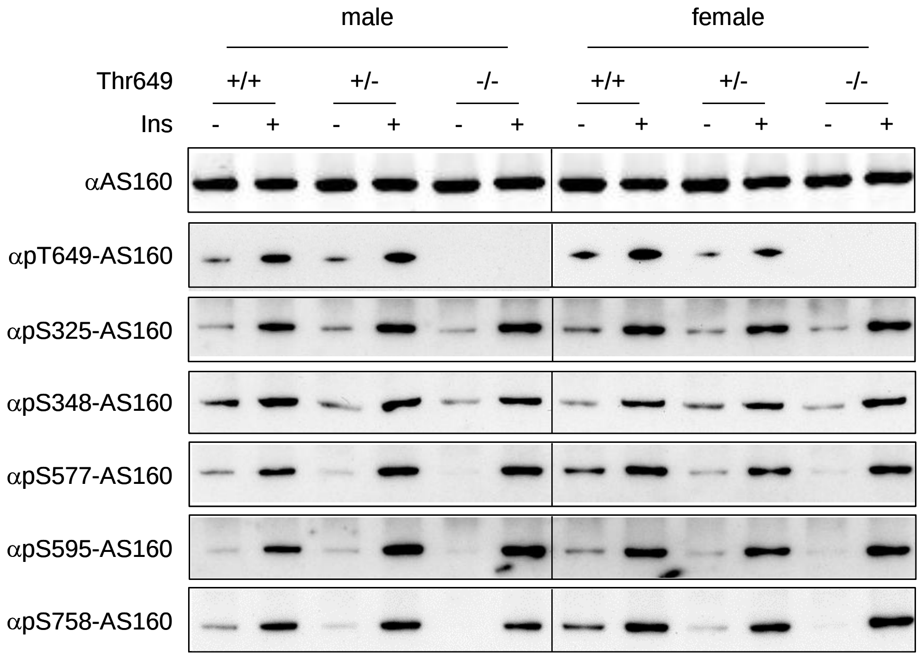

Figure S2B

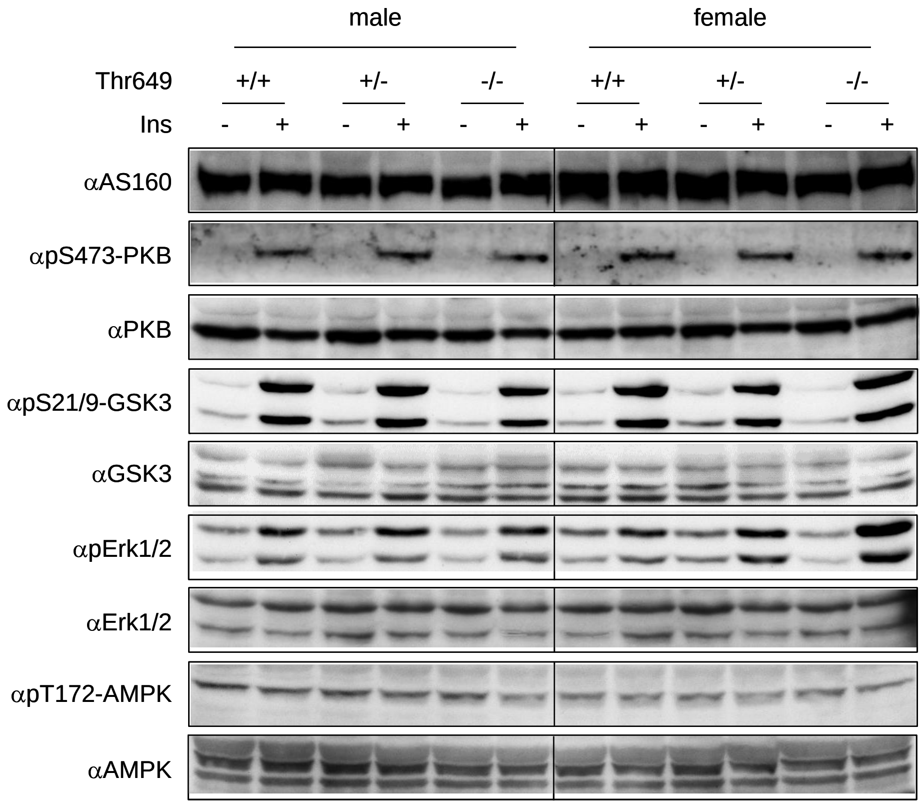

Figure S3

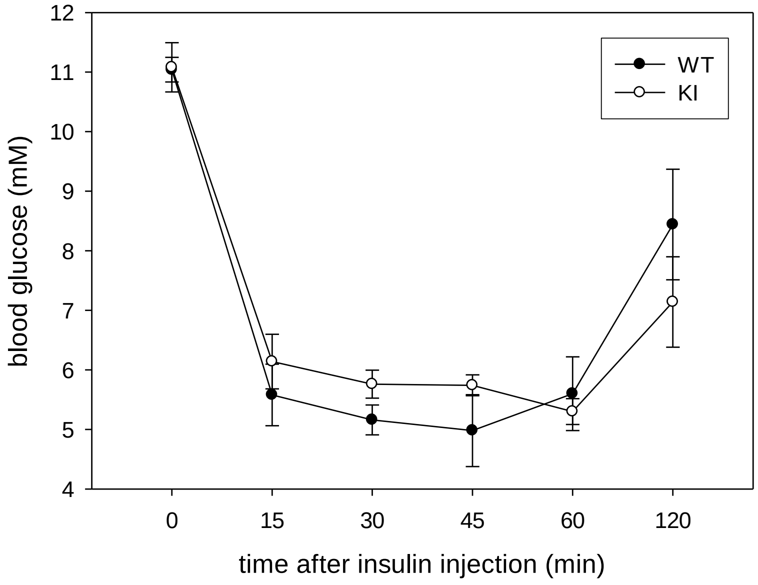

12
11
10
9
8
7
6
5
4
blood glucose (mM)
WT
KI
0
15
30
45
60
120
time after insulin injection (min)

Figure S4A

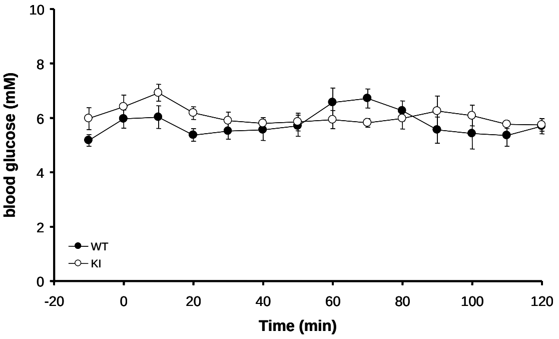

Figure S4B

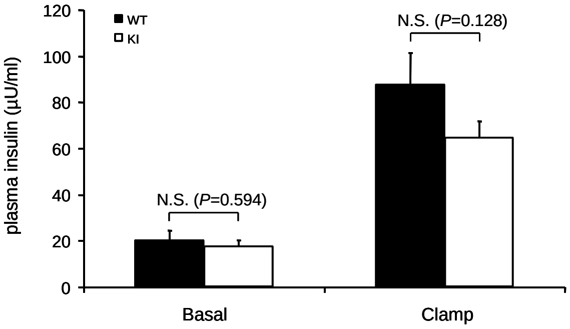

Figure S4C

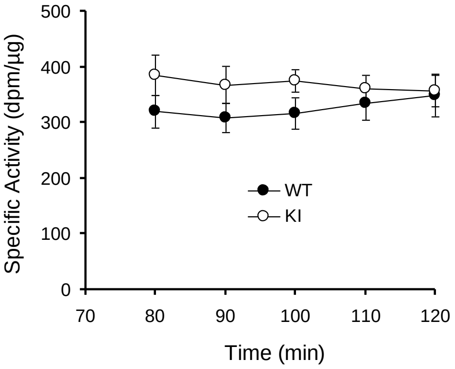

Figure S4D

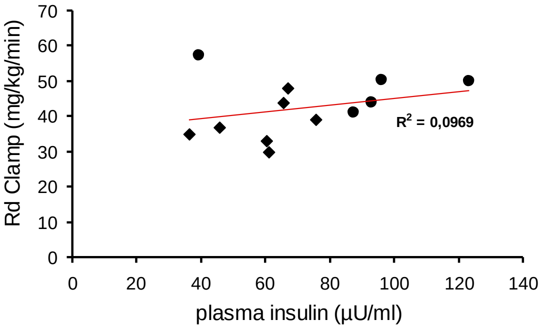

Figure S4E

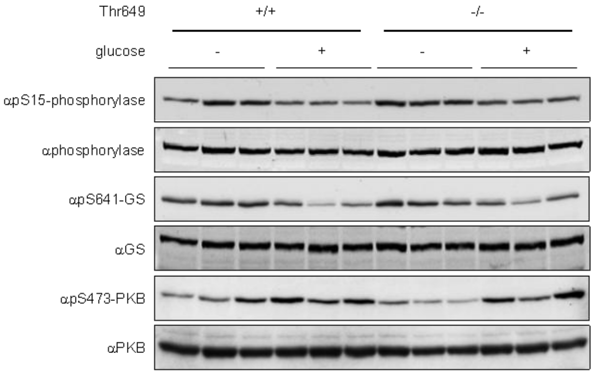

Figure S4F

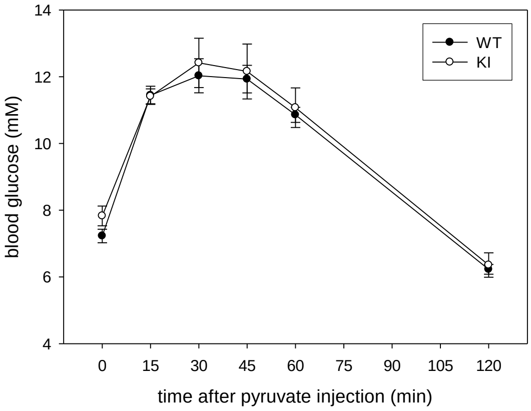

Figure S5A

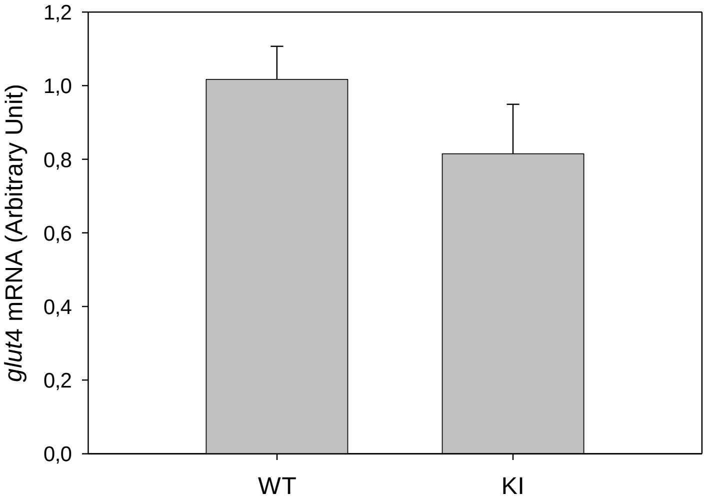

Figure S5B

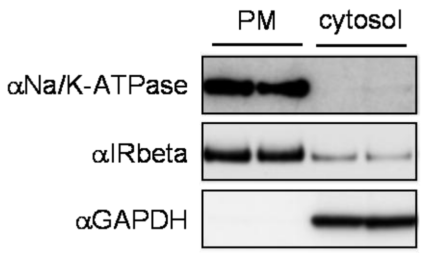

Figure S1, Related to Figure 1. Generation and Basic Characterisation of Mice with AS160 Thr649Ala Knockin Mutation

A. Genotyping PCR analysis of wild-type and heterozygous/homozygous knock-in AS160 alleles. The PCR fragments are 287 basepairs (bp) for the wild-type allele and 408 bp for the knock-in allele.

B. Tissue expression of total AS160 protein levels in the AS160 knock-in mice and wild-type littermates. The wild-type, heterozygous and homozygous AS160 Thr649Ala knock-in male mice (8-week-old) were anaesthetized and injected with either saline buffer or insulin (150 mU/g body weight) for 20 min before the respective tissues were removed. AS160 protein levels were determined by immunoblot analysis in 40 µg of total lysates from each tissue.

C. Growth of the wild-type and AS160 Thr649Ala knock-in male mice. Body weight was monitored weekly in both wild-type and AS160 knock-in male mice (wt, n=24; KI, n=23).

D. Growth of the wild-type and AS160 Thr649Ala knock-in female mice. Body weight was monitored weekly in both wild-type and AS160 knock-in female mice (wt, n=18; KI, n=32). * indicates $P<0.05$ (t-test).

E. Body composition of AS160 Thr649Ala knock-in mice. Organs from the wild-type and AS160 Thr649Ala knock-in mice (6-month-old) were removed and weighed. The data are given as the mean ± SEM (n=7 for each genotype). * indicates $P<0.05$ (t-test).

Figure S2, Related to Figure 2. Insulin-Stimulated Phosphorylation of AS160, PKB, GSK3, Erk1/2, and AMPK in Hearts from the Wild-Type and AS160 Knockin Mice

The wild-type, heterozygous and homozygous AS160 knock-in mice (8-week-old) were anaesthetized and injected with either saline buffer or insulin (150 mU/g body weight) for 20 min before the respective tissues were removed.

A. Insulin-stimulated phosphorylation of AS160. AS160 proteins were immunoprecipitated from 0.5 mg of heart lysates, and the phosphorylation of AS160 on Ser325, Ser348, Ser577, Ser595, Thr649 and Ser758 was determined by immunoblotting using the respective phospho-specific antibodies.

B. The total and phosphorylated forms of PKB, GSK3, Erk1/2 and AMPK were determined by immunoblotting in 40 µg of heart lysates using the respective phospho-specific antibodies or total antibodies indicated in the Experimental Procedures. Total AS160 protein was used as a loading control.

Figure S3, Related to Figure 3. Insulin Tolerance Test of AS160 Knockin and Wild-Type Mice

Male wild-type and AS160 knock-in mice (8 to 10-week-old) were subject to intraperitoneal injection with insulin (0.75 mU/g body weight) after restriction from food for 5 h. At time zero, and the indicated times after injection, blood glucose levels were determined with a Breeze 2 glucometer (Bayer). The data are given as the mean ± SEM (n=5 for each genotype).

Figure S4, Related To Figure 4. Blood Glucose and Insulin Levels during Hyperinsulinemic-Euglycemic Clamp, Phosphorylation of Key Enzymes in Liver, and Pyruvate Tolerance Test

A. Blood glucose levels during hyperinsulinemic-euglycemic clamp.

B. Insulin levels in the plasma before and at the end of hyperinsulinemic-euglycemic clamp. N.S., not significant.

C. Plasma glucose specific activity during t=80 to 120 min of the hyperinsulinemic, euglycemic clamp period. The slope of the relationship between glucose specific activity and time was not significantly different from 0, demonstrating that this variable was in a steady state.

D. The whole body glucose disappearance rates (Rd) at the end of insulin clamp were plotted against plasma insulin levels at the end of 120 min infusion in individual wild-type (●) and AS160 knock-in (◆) mouse. Red line shows the regression line (R^2=0.0969).

E. Phosphorylation of glycogen phosphorylase, GS and PKB in the liver from male mice (10 to 12-week-old) after intraperitoneal injection with glucose. The wild-type and AS160 knock-in mice were injected with glucose (2 mg/g body weight) for 20 min before livers were removed. The phosphorylation of glycogen phosphorylase, GS and PKB, and the total glycogen phosphorylase, GS and PKB were determined in 40 μg of liver lysates using the respective phospho-specific antibodies and total antibodies indicated in Experimental Procedures.

F. Pyruvate tolerance test of AS160 knock-in and wild-type male mice at 9 to 11 weeks of age. Prior to pyruvate administration, mice were fasted for 16 h to achieve a baseline blood glucose level. At time zero, and the indicated times after intraperitoneal injection of pyruvate (2 mg/g body weight), blood glucose levels were determined with a Breeze 2 glucometer (Bayer). The data are given as the mean ± SEM (n=7 to 8 for each genotype).

Figure S5, Related to Figure 6. *glut*4 Transcripts in Heart and Plasma Membrane Bound GLUT4 in Primary Adipocytes

A. The *glut*4 mRNA levels of in the wild-type and AS160 Thr649Ala knock-in mice. Total RNA was extracted from hearts of male mice (20-week-old) and the *glut*4 mRNA levels were determined via quantitative RT-PCR. The values represent the mean (± SEM) from five mice for each genotype.

B. Markers for fractionation of adipose. Epididymal fat pads were removed from male mice and subject to membrane fractionation. The plasma membrane fractions were separated from the intracellular fractions containing GLUT4 vesicles. Na^+/K^+-ATPase alpha-1 and insulin receptor beta (IRbeta) were used as plasma membrane markers, and GAPDH as a cytosol marker.

Supplemental Experimental Procedures

Materials

Recombinant human insulin was from Novo Nordisk (Denmark) and glucose from Baxter Clintec (UK). Microcystin-LR was from Linda Lawton (Robert Gordon's University, UK), and protease-inhibitor cocktail tablets from Roche. 2-deoxy-D-[1-^{3}H]glucose and ^{14}C-mannitol were from Amersham Pharmacia Biotech (Little Chalfont, UK). The biotinylated photolabel Bio-LC-ATB-BGPA was kindly provided by Professor Geoffrey Holman (University of Bath, UK). All other chemicals were from BDH Chemicals or Sigma-Aldrich.

Antibodies

Sheep antibodies against AS160 and TBC1D1 were as previously described (Chen et al., 2008; Geraghty et al., 2007). The pan-14-3-3 antibody was K-19 from Santa Cruz. Antibodies that recognise phosphorylated Thr172 on AMPK, phosphorylated Thr308 and Ser473 on PKB, phosphorylated Thr202/Tyr204 on Erk1/2, phosphorylated Ser21/9 on GSK3α/β, anti-AMPK, anti-PKB and anti-Erk1/2 were from Cell Signaling Technology. The antibody that recognises total GSK3α/β was from Upstate, and GLUT4 antibody (ab654) was from Abcam.

Generation of Conditional Thr649Ala-AS160/TBC1D4 Knockin mice

By following the knock-in strategy outlined in Fig 1, TaconicArtemis (Cologne, Germany) generated heterozygous Thr649Ala-AS160/TBC1D4 knock-in mice (C57BL/6 genetic background).

Mice Breeding and Genotype Analysis

In Dundee, all animal studies and breeding were approved by the University of Dundee Ethics Committee and performed under a United Kingdom Home Office project license. AS160 knock-in and wild-type mice were housed with a light/dark cycle of 12 h, and free access to food and water unless stated. Genotyping was performed by PCR, and the presence of a wild-type or Thr649Ala knock-in allele was detected using two primers, 5'-ATCTTGGGGCACTATCAACC-3' and 5'-CAGTGGCATGATCTCTGTGG-3'.

Tissue Lysis and Immunoprecipitations

Unless stated, mouse tissues were homogenized with a Polytron tissue processor (Glen Mills Inc.) in ice-cold lysis buffer (50 mM Tris-HCl (pH 7.4), 1 mM EDTA, 1 mM EGTA, 1% (v/v) Triton X-100, 1 mM sodium *ortho*-vanadate, 10 mM sodium glycerophosphate, 50 mM sodium fluoride 5 mM sodium pyrophosphate, 0.27 M sucrose, 2 μM microcystin-LR, 1 mM benzamidine, 0.1% (v/v) 2-mercaptoethanol and complete proteinase inhibitor mixture (Roche). Lysates were clarified by centrifugation, snap frozen in liquid nitrogen and stored at -80°C until analysis. Protein concentrations were determined with Bradford reagent (Thermo).

The lysates were incubated with antibody-coupled protein G-Sepharose overnight at 4°C. The suspension was centrifuged to sediment between washes. The immunoprecipitates were extracted in SDS sample buffer.

Western Blot and Quantitation

After SDS-PAGE, proteins were transferred onto nitrocellulose membranes and immunoblotted using the indicated primary antibodies. Detection was performed using horseradish-peroxidase-conjugated secondary antibodies (Promega) and ECL® (enhanced chemiluminescence reagent; Amersham Biosciences). The images were scanned, imported into a LI-COR Odyssey imaging system and quantified.

Plasma Insulin, Adiponectin, Leptin, and Free Fatty Acid Measurements

Blood was collected from tail veins of random-fed or overnight fasted mice. Plasma insulin, adiponectin and leptin levels were determined using a rat/mouse insulin ELISA kit, adiponectin ELISA kit, and mouse leptin ELISA kit, respectively (Linco Research). Plasma free fatty acid levels were determined using a Wako NEFA C test kit (Wako Chemicals USA, Inc.).

Intraperitoneal Glucose Tolerance Test, Insulin Tolerance Test, and Pyruvate Tolerance Test

Mice were deprived of food for 5 hours (insulin tolerance test) or overnight (16 h, glucose and pyruvate tolerance test), and basal blood glucose was measured using a Breeze 2 glucometer (Bayer)

following tail incision. The mice were administered 2 mg/g body weight glucose or pyruvate, or 0.75 mU/g body weight insulin by i.p. injection, and blood glucose measured at the indicated times.

Subcellular Fractionation of Adipose and Soleus

Subcellular fractionation was carried out as previously described (Larance et al., 2005) with some modifications. Briefly, adipose or soleus was homogenized in the same lysis buffer as above except that detergent was omitted. The homogenates were centrifuged at 500 x g for 10 min to remove unbroken cells. The supernatants were further centrifuged at 10, 000 x g for 12 min to obtain crude plasma membrane (PM) together with nucleus and mitochondria, and intracellular fraction containing cytoplasm and the GLUT4 storage vesicles. The PM fraction was solubilized in detergent-containing (1% v/v Triton) lysis buffer.

Statistical Analysis

Unless stated, data analysis was performed via ANOVA (SigmaStat) with Holm-Sidak method for post-test, and differences were considered statistically significant at $P<0.05$.
